# Evaluation of the relationship between end-tidal carbon dioxide level and heart failure classification

**DOI:** 10.1590/1806-9282.20231499

**Published:** 2024-05-20

**Authors:** Murat Tepe, Hakan Hakkoymaz, Ali İhsan Kilci, Muhammed Semih Gedik, Cebrail Öztürk, Mehmet Kubilay Gökçe, Ahmet Çağrı Aykan

**Affiliations:** 1 Necip Fazıl City Hospital, Emergency Medicine – Kahramanmaraş, Turkey.; 2 Kahramanmaraş Sütçü İmam University, Emergency Medicine – Kahramanmaraş, Turkey.; 3 Kahramanmaraş Sütçü İmam University, Cardiology – Kahramanmaraş, Turkey.

**Keywords:** Emergency medicine, Heart failure, Capnography

## Abstract

**OBJECTIVE:**

Heart failure is a disease with cardiac dysfunction, and its morbidity and mortality are associated with the degree of dysfunction. The New York Heart Association classifies the heart failure stages based on the severity of symptoms and physical activity. End-tidal carbon dioxide refers to the level of carbon dioxide that a person exhales with each breath. End-tidal carbon dioxide levels can be used in many clinical conditions such as heart failure, asthma, and chronic obstructive pulmonary disease. The aim of the study was to reveal the relationship between end-tidal carbon dioxide levels and the New York Heart Association classification of heart failure stages.

**METHODS:**

This study was conducted at Kahramanmaraş Sütçü İmam University Faculty of Medicine Adult Emergency Department between 01/03/2019 and 01/09/2019. A total of 80 patients who presented to the emergency department with a history of heart failure or were diagnosed with heart failure during admission were grouped according to the New York Heart Association classification of heart failure stages. The laboratory parameters, ejection fraction values, and end-tidal carbon dioxide levels of the patients were measured and recorded in the study forms.

**RESULTS:**

End-tidal carbon dioxide levels and ejection fraction values were found to be significantly lower in the stage 4 group compared to the other groups. Furthermore, pro-B-type natriuretic peptide (BNP) values were found to be significantly higher in stage 4 group compared to the other groups.

**CONCLUSION:**

It was concluded that end-tidal carbon dioxide levels could be used together with pro-BNP and ejection fraction values in determining the severity of heart failure.

## INTRODUCTION

Heart failure (HF) is clinically defined as a clinical syndrome in which patients experience typical symptoms such as dyspnea, swelling of the legs, fatigue, and lung rales. Additionally, the cardiac apex beat is displaced, and there is an increase in jugular venous distension during inspection^1^.

There are different classifications of heart failure, such as systolic/diastolic heart failure, acute/chronic heart failure, right/left heart failure, and high/low output heart failure^2^. The most commonly used classification system is the one established by the New York Heart Association (NYHA), which evaluates the effect of HF on the patient’s physical capacity^1^.

The change in carbon dioxide (CO_2_) level over time can be evaluated by CO_2_ waveform or capnography. End-tidal carbon dioxide (EtCO_2_) is the level of CO_2_ that a person exhales with each breath^3^ and can be used to determine the prognosis of HF patients, along with the NYHA classification and left ventricular ejection fraction (EF)^4^.

The aim of this study was to reveal the relationship between the level of EtCO_2_, which is considered to be effective in the clinical course and prognosis of HF, and the NYHA classification of heart failure.

## METHODS

In this study, patients who presented to the adult emergency department between March 01, 2019 and September 01, 2019 with a history of HF or a diagnosis of HF at admission were grouped according to NYHA stages. A total of 80 patients, including 20 patients with NYHA stage 1, 20 patients with stage 2, 20 patients with stage 3, and 20 patients with stage 4, were included in the study. Ethical approval was obtained from the Ministry of Health of the Republic of Turkey and the Ethics Committee of Kahramanmaraş Sütçü İmam University Faculty of Medicine with resolution number 23 in session 2019/03 on February 20, 2019. Written informed consent was obtained from all patients included in our study.

Voluntary patients older than 18 years of age who had a diagnosis of HF or were newly diagnosed with HF were included in the study. Those with pathologies that may affect EtCO_2_ values such as sepsis and septic shock, hemorrhagic shock, pulmonary embolism, pneumothorax, renal failure, acute cerebrovascular accident, chronic obstructive pulmonary disease (COPD), asthma, drug intoxication, and metabolic acidosis–alkalosis were excluded from the study. Patients’ demographic data, medical history, and sampling dates were recorded on the previously prepared study forms.

N-terminus pro-B-type natriuretic peptide (NT pro-BNP) analysis was evaluated with the AQT 90 flex (Radiometer Medical Aps, Bronshoj, Denmark) device. EtCO_2_ levels of the patients were measured by the sidestream method using the Capnostream^®^ 20 (Oridion, Jerusalem, Israel) monitor and disposable nasal cannula. The echocardiographic examinations of the patients included in the study were performed by cardiology clinic physicians using the GE Vivid S5 (General Electric Ving Med Systems, Horten, Norway) brand device. The EF values were calculated using the modified Simpson method.

In the evaluation of the data, the conformity of the variables to the normal distribution was examined by the Shapiro-Wilk test. Group comparisons of normally distributed variables were analyzed by one-way analysis of variance. Tukey HSD, one of the multiple comparison tests (post hoc), was used. Group comparisons in non-normally distributed variables were analyzed using the Kruskal-Wallis H test. Multiple comparisons were analyzed by the Dunn-Sidak test and Bonferroni test. The relationship between group distributions in categorical variables was examined by chi-square test and exact test. Statistical significance was considered p<0.05. The data were evaluated in the IBM SPSS version 22 program.

## RESULTS

A total of 80 patients who had a diagnosis of heart failure or were newly diagnosed with heart failure, consisting of 20 patients each from stage 1, stage 2, stage 3, and stage 4 according to the NYHA classification, were included in our study. Blood tests were taken from each patient included in the study, echocardiography was performed by the cardiology department, and the EtCO_2_ values were measured. Patients consisted of 52 (65%) males and 28 (35%) females.

The mean age was found to be 62.75 years for the patients in the stage 1 group, 68.90 years for the patients in the stage 2 group, 70.90 years for the patients in the stage 3 group, and 73.50 years for the patients in the stage 4 group. The mean age of the stage 4 group was significantly higher compared to the stage 1 group, which was found to be statistically significant (p<0.05). When the medical histories of the patients were examined, 28 (35%) of them had diabetes mellitus, 55 (68.75%) had hypertension, and 56 (70%) had coronary artery disease. No significant difference was found between the groups in terms of hypertension, coronary artery disease (CAD), diabetes mellitus (DM), and malignancy. The distribution of age and comorbidities by stages is presented in Table 1.

**Table 1:** Distribution of age and comorbidities by stages.

			Stage				p
			1	2	3	4	
Age		Mean±SD	62.75±13.31^4^	68.90±7.88	70.90±11.49	73.50±14.76^1^	0.043*
Diabetes mellitus	No	n (%)	13 (65,0)	11 (55,0)	13 (65,0)	15 (75,0)	0.624
	Yes	n (%)	7 (35,0)	9 (45,0)	7 (35,0)	5 (25,0)	
Hypertension	No	n (%)	10 (50,0)	4 (20,0)	6 (30,0)	5 (25,0)	0.185
	Yes	n (%)	10 (50,0)	16 (80,0)	14 (70,0)	15 (75,0)	
Coronary artery disease	No	n (%)	2 (10,0)	9 (45,0)	7 (35,0)	6 (30,0)	0.103
	Yes	n (%)	18 (90,0)	11 (55,0)	13 (65,0)	14 (70,0)	

* Statistically significant p-value.

When the NT pro-BNP values were examined, the pro-BNP values in the stage 2 and stage 4 groups were found to be significantly higher compared to the stage 1 and stage 3 groups (p=0.001).

When the EF (%) of the patients included in the study was evaluated, the mean EF (%) values of the patients within the group were found to be 40.75% in the stage 1 group, 34.50% in the stage 2 group, 33.15% in the stage 3 group, and 30.25% in the stage 4 group. The EF values were found to be statistically significantly lower in the stage 4 group compared to the stage 1 group (p=0.013).

When the groups were examined in terms of EtCO_2_ values, the mean EtCO_2_ values of the patients within the group were found to be 32.45 in the stage 1 group, 31.30 in the stage 2 group, 33.15 in the stage 3 group, and 25.75 in the stage 4 group. The EtCO_2_ values were found to be significantly lower in patients in the stage 4 group compared to the other groups (p<0.001).

Patients’ mean EF and EtCO_2_ values and median NT pro-BNP values by stages are presented in Table 2.

**Table 2:** Distribution of laboratory, ejection fraction, and end-tidal carbon dioxide values by stages.

		Stage 1	Stage 2	Stage 3	Stage 4	p
NT pro-BNP	Median	1,305.00^2,4^	5,775.00^1,3,4^	2,412.50^2,4^	6,930.00^1,3^	0.001*
EF	Mean	40.75^4^	34.50	32.75	30.25^1^	0.013*
EtCO_2_	Mean	32.45^4^	31.30^4^	33.15^4^	25.75^1,2,3^	<0.001*

* Statistically significant p-value.

The superscript numbers indicate New York Heart Association Functional Classification Stages.

## DISCUSSION

The level of EtCO_2_ provides important information about the current clinical and respiratory status of the patient^5^. It is used in many clinical situations, which are cardiac arrest, confirmation of endotracheal intubation tube location, procedural sedation, trauma, sepsis, metabolic acidosis, pulmonary embolism, heart failure, respiratory distress, and convulsions^6^.

In the study of Glöckner et al., when the gender distribution of the patients was examined, the rate of male patients was found to be 68.0%^7^; however, this rate was found to be 61.2%^8^ as a result of a study conducted by Roger et al. Notably, 65% of the patients included in our study were male. This rate we found in our study is similar to other studies in the literature.

Considering the comorbidities of the patients included in the study, it was determined that while 35% of them had DM, 68.75% had HT, and 70% had CAD. In a study conducted by Roger et al., it was determined that 88 and 76% of the patients had HT and CAD, respectively^8^. In most of the studies in the literature, HT and CAD were found in the first place among comorbidities. Our study is also similar to other studies in the literature. In our study, when comorbidities were examined by stages, no statistically significant difference was found between the groups in terms of HT, DM, and CAD. Similar to our study, in the study conducted by Williams et al.^9^, no statistically significant difference was found between the groups in terms of HT, DM, and CAD by stages.

Natriuretic peptides (NP) play an important role in diagnosing HF. Especially low levels of NP are very valuable to exclude the diagnosis of HF^10^. In our study, we examined the pro-BNP values of our patients by stages and found that the median values were the lowest at 1,305 pg/mL in the stage 1 group and the highest at 6,930 pg/mL in the stage 4 group. The difference between the groups was statistically significant. In the study conducted by Baggish et al., the pro-BNP median values by stages were 3,512 pg/mL in the stage 2 group, 5,610 pg/mL in the stage 3 group, and 6,196 pg/mL in the stage 4 group, and the difference between the stages was also found to be significant^11^. Similarly, in a study conducted by Arat-Özkan et al., it was found that pro-BNP levels increased as the stage progressed, which was statistically significant^12^. In our study, the fact that the pro-BNP values were the highest in the stage 4 group and the lowest in the stage 1 group was similar to the literature.

The EF (%) of the patients in our study were measured and compared by stages. The mean EF (%) values were found to be 40.75 in the stage 1 group, 34.50 in the stage 2 group, 32.75 in the stage 3 group, and 30.25 in the stage 4 group, and the difference between the groups was statistically significant. In a study conducted by Hu Ying et al., it was determined that EF decreased as the stage progressed, and the difference between the stages was found to be significant^13^. In most of the data in the literature, it was determined that EF values decreased as the NYHA stage progressed, and a similar result was found in our study.

It has been reported that the evaluation of EtCO_2_ measurement with capnography can be used together with NYHA staging in the prognosis of HF4. The EtCO_2_ values of the patients included in our study were measured, and when we examined them by stages, they were found to be significantly lower in the stage 4 group compared to the other groups. The results were found to be similar between stage 1, 2, and 3 groups. In a study conducted by Seguchi et al., it was determined that EtCO_2_ values decreased as the stage progressed, which was statistically significant; similar to our study, the lowest values were found in the stage 4 group in that study^14^. In the study conducted by Matsumoto et al., the EtCO_2_ levels were compared between the NYHA stage 1, 2, and 3 groups and the control group consisting of healthy subjects. A significant decrease was found in EtCO_2_ levels as the NYHA stage progressed, and the EtCO_2_ values of the control group were found to be significantly higher compared to the group consisting of patients^15^. In the study conducted by Tanabe et al., patients in NYHA stage 1, 2, and 3 groups were compared, and it was determined that the EtCO_2_ levels decreased as the stage progressed^16^. Unlike the literature, no significant difference was found between stage 1, 2, and 3 groups in our study. Furthermore, patients with pathologies such as sepsis and septic shock, hemorrhagic shock, pulmonary embolism, pneumothorax, renal failure, acute cerebrovascular accident, COPD, asthma drug intoxication, and metabolic acidosis–alkalosis that may affect EtCO_2_ values were excluded from our study. Thus, this is the first study in the literature.

## CONCLUSION

The incidence of heart failure increases in direct proportion to the prolongation of life expectancy due to advancing medical treatment possibilities, which increases the admission of patients with HF to emergency departments.

In this study, the EtCO_2_ values measured in patients with heart failure were found to be significantly lower in NYHA stage 4 patients. We believe that the measurement of EtCO_2_ values in patients with HF can be used to determine the severity of HF.
